# Genetic evaluation of early growth in horro sheep under smallholder farm management, Oromia Regional State, Ethiopia

**DOI:** 10.1371/journal.pone.0347868

**Published:** 2026-05-08

**Authors:** Tamiru Kibebew, Tesfaye Getachew, Kassahun Bekana Kitila, Gemeda Duguma Jaleta

**Affiliations:** 1 Buno Bedele Zone Agricultural and Natural Resource Office, Buno Bedele Zone, Oromia, Ethiopia; 2 The International Center for Agricultural Research in the Dry Areas (ICARDA), Addis Ababa, Ethiopia; 3 Ambo University, Guder Mamo Mezemir Campus, Guder, Ethiopia; 4 Northwest A & F University, Xianyang, China; 5 School of Veterinary Medicine, Wallaga University, Nekemte, Ethiopia; Ain Shams University Faculty of Agriculture, EGYPT

## Abstract

The study was conducted under on-farm management conditions in the Horro district of the Horro Guduru Wallaga zone in Oromia region. Flocks from the Community-Based Breeding Program (CBBP) were used for this study, which aimed to estimate genetic parameters for early growth traits. A total of 2,480 birth weight records, 2,441 three-month weight records, and 770 six-month weight records were analyzed. Least-squares means (LSM) analysis was performed using the General Linear Model (GLM) procedures of the Statistical Analysis System (SAS) to determine the effects of fixed factors. To estimate the (co) variance components and genetic parameters for growth traits, Restricted Maximum Likelihood (REML) procedures were implemented in the VCE 4.2.5 package. Multivariate genetic analysis was employed to estimate genetic correlations among traits. The least-squares means for birth weight (BWt), three-month weight (3MWt), and six-month weight (6MWt) were 2.72 ± 0.01, 12.33 ± 0.03, and 16.57 ± 0.07, respectively. The estimates of direct heritability obtained from the animal model were 0.16 ± 0.01, 0.19 ± 0.02, and 0.28 ± 0.35 for BWt, 3MWt, and 6MWt, respectively. The genetic correlations between BWt and 3MWt, BWt and 6MWt, and 3MWt and 6MWt were 0.30, 0.03, and 0.11, respectively. The moderate heritability estimates obtained for the growth traits indicate that genetic improvement through selection could lead to meaningful genetic change.

## Introduction

Sheep are an important species of livestock in Ethiopia, with an estimated population of 42.9 million, of which about 42.7 million are indigenous breeds and 174,559 are hybrids [1]. Nine sheep breeds have been identified so far using both phenotypic and genetic characterization [2]. The various indigenous sheep breeds in the country are owned and managed by resource-poor farmers and pastoralists, who keep the animals under traditional management practices and extensive production systems [3]. Literature suggests that sheep play a significant role in the country’s economic development and the livelihoods of farmers and pastoralists; however, their productivity remains low due to several factors. Major constraints reported include weak genetic improvement efforts stemming from unclear breeding strategies and policies, feed shortages, inadequate extension services or lack of improved technologies, diseases and parasites, poor infrastructure, and insufficient market information [4–6].

Various efforts have been made in the past to improve sheep production and productivity in Ethiopia [4]. These include the characterization of indigenous sheep breeds, genetic improvement through an open nucleus breeding system, and crossbreeding with exotic breeds. However, the involvement of producers, owners, and relevant stakeholders in these efforts has been minimal. The limited participation of stakeholders, particularly smallholder farmers and pastoralists, in the planning and implementation of sheep improvement programs may have contributed to these failures. Recognizing this issue, the International Livestock Research Institute (ILRI), the International Center for Agricultural Research in Dry Areas (ICARDA), and the Austrian University of Natural Resources and Life Sciences (BOKU), in collaboration with the Ethiopian National and Regional Agricultural Research Systems (NARS), designed and implemented Community-based Sheep Breeding Programs (CBBPs) in various areas of the country, funded by the Austrian Development Agency from 2007 to 2011 [3,5,7]. The implementation of CBBPs began in 2009 for four sheep breeds (Menz, Afar, Horro, and Bonga) across four different regions in Ethiopia. Farmers and stakeholders were involved in all stages of the design and implementation of the CBBPs. The initiated CBBP has been successful and is currently ongoing, except in Afar, primarily due to the mobility of sheep producers in pastoral areas searching for feed and water. This mobility has made it difficult to manage the breeding program effectively [8].

The main objective of the CBBPs was to improve various sheep breeds for growth and reproduction traits through selective breeding. For effective genetic evaluation and selection, it is essential to estimate variance-covariance components and genetic parameters for economically important traits at the appropriate time. Estimating genetic parameters such as heritability and genetic correlations among traits is vital for designing suitable breeding programs aimed at maximizing genetic improvement. These estimates indicate the relative genetic importance of traits, either as direct genetic responses or as correlated responses to selection [9]. Therefore, this study aims to estimate and evaluate the genetic parameters of early growth in the Horro sheep breed, which is managed under a community-based breeding program in the Horro district.

## Materials and methods

### Description of the study area

The study focused on flocks of the Horro sheep breed owned by participants in the ongoing community-based breeding program. This program was initiated in 2009 in the Gitilo and Lakku Igu villages of the Horro district. The Horro district is located in the Horro Guduru Wallaga zone of the Oromia Regional State, approximately 315 km northwest of Addis Ababa. Its geographical coordinates are 9°34′ N latitude and 37°06′ E longitude. The area’s production system is mixed crop-livestock. The district experiences a long rainy season that lasts from March to mid-October, with an average annual precipitation of about 1800 mm and maximum and minimum temperatures of approximately 22.67°C and 11.75°C, respectively. The Horro district comprises eleven kebeles (villages), with the community-based breeding program initiated in two: Gitilo and Lakku Igu. Gitilo is located about 12 km, and Lakku Igu is about 7 km from Shambu, the capital of the Horro Guduru Wallaga zone [10].

### Description of the breed and flock management

The Horro sheep breed is one of nine sheep breeds identified in Ethiopia. It derives its name from the Horro region, which is the primary natural habitat of the breed. Although Horro is recognized as a place name, it also refers to one of the renowned Oromo tribes in the Horro Guduru Wallaga zone. The Horro sheep breed is widely distributed throughout much of the western part of the country, located between 35–38°E and 6–10°N. The breed characteristics was well described by [11] and [12].

Farmers maintain their sheep flocks together in communal grazing areas during the day and separate them at night. Most farmers have constructed flock houses adjacent to their kitchens for nighttime shelter. The primary feed sources in the study area consist of natural pasture and aftermath grazing. Occasionally, some farmers provide supplemental feeds, such as local brewery by-products known as atela and salt, for pregnant ewes, breeding rams, and castrated rams. Since the initiation of the Community-Based Breeding Program, all sheep participating in the program have been ear tagged. Regular record-keeping of birth dates, birth weights, three-month weights, six-month weights, and yearling weights is conducted in both Gitilo and Laku Igu villages by hired enumerators [10].

### Data collection and statistical analysis

Data collected from 2010 to 2016 were obtained from the Bako Agricultural Research Centre (BARC). The data were recorded by hired enumerators in two villages, Gitilo and Laku Igu, under the supervision of BARC. During the data cleaning process, birth weight (BWt), three-month weight (3MWt), and six-month weight (6MWt) records were included, ensuring the fixed effects of year of birth, season of birth, sex, and location/village were accounted for. The seasons were categorized into four major groups: early rain, wet, early dry, and dry. While processing the data, efforts were made to incorporate the lambs’ maternal and paternal information; however, the data proved inconsistent. Therefore, it is unable to include the maternal effect in the model. Following the data cleaning, the final analysis included 2,480 records of BWt, 2,441 records of 3MWt, and 770 records of 6MWt.

The General Linear Model (GLM) procedure of the Statistical Analysis System (SAS, 2016, version 9.4) was employed to examine the fixed effects of season, year, location, and sex. This model was utilized to estimate the growth performances of BWt, 3MWt, and 6MWt.

The statistical model fitted was:


$${\text{Y}}_{\text{ijkl}}\;=\;\mu +{\text{G}}_{\text{i}}\;+\;{\text{Y}}_{\text{j}}\;+\;{\text{S}}_{\text{k}}\;+\;{\text{L}}_{\text{l}}+\;{\text{e}}_{\text{ijk}}$$

Where:

Y_ijkl_= the observation on BWt, 3MWt, and 6MWt

μ = overall mean

G_i_ = fixed effect of i^th^ Sex (male, female)

Y_j_ = fixed effect of j^th^ birth year (2010 … 2016)

S_k_ = fixed effect of k^th^ birth season (early-rain, wet, early-dry, dry)

L_l_ = fixed effect of l^th^ location (l = 2, Gitilo, Laku Igu)

e_ijkl_ = effect of random error

The (co)variance components and genetic parameter estimation (heritability, genetic, and phenotypic correlations) were analyzed by fitting a multivariate animal model using VCE 6.0 and PESTE 4.2.5, employing the restricted maximum likelihood method [13].

The simple animal model fitted was as follows:


Y = Xb + Za + e

Where Y is the vector of records,

X = fixed effects

b = s a vector of fixed effects (including the overall mean)

Z = random additive genetic effects

a = is a vector of random additive genetic effects and

e = a vector of random errors.

This model was utilized due to the limited availability of maternal information within the dataset. Additionally, there are deficiencies in the record-keeping of the animals’ pedigree structures. Enumerators primarily concentrated on recording birth weight, six-month weight, and yearling body weight, often neglecting to accurately document the dam and sire information.

### Ethical statement

The data used in this study were collected from 2010 to 2016. This research does not involve any impact on animal welfare, nor does it require contact with animals. Therefore, we, the authors, declare that no ethical approval is necessary for this research.

## Results and discussion

### Early growth performance

#### Birth weight (BWt).

All fixed effects considered had significant impacts (p < 0.05) on birth weight, except for the season of birth. The overall mean birth weight in the current study was 2.72 ± 0.01 kg (Table 1). It aligns with the analyzed data from 2009 to 2018, which indicates an average birth weight of 2.80 ± 0.70 kg for the same breed under community-based breeding [10]. Moreover, it also closely aligns with the 2.71 kg average birth weight reported by [14], who analyzed data from 1977 to 1998 for the same breed at Bako Agricultural Research Center. However, it is higher than the 2.6 kg reported by Alemayehu et al. [15], who examined data from 1978 to 2011 for Horro sheep at the same research center. The differences in birth weight may be attributed to location and the size of the data collection period. The current study was conducted in the Horro highlands (altitude range 2170–2853 m.a.s.l.), believed to be the breed’s natural habitat, while the flocks at Bako Agricultural Research Center are situated in the warm, humid midlands (altitude range 1579–1789 m.a.s.l.) [10].

**Table 1:** Least square mean of fixed factors affecting growth traits of Horro sheep.

Effect	BWt (kg)		3MWt (kg)		6MWt (kg)	
	N	LSM ± SE	N	LSM ± SE	N	LSM ± SE
Overall	2480	2.72 ± 0.01	2441	12.33 ± 0.03	770	16.57 ± 0.07
**Sex**	**2480**		**2441**		**770**	
Male	1222	2.76 ± 0.02^a^	1203	12.24 ± 0.05	419	16.80 ± 0.11
Female	1258	2.68 ± 0.02^b^	1238	12.25 ± 0.05	351	16.58 ± 0.10
*p-value*		*0.0001*		*0.9260*		*0.10640*
**Location**	**2480**		**2441**		**770**	
Laku	807	2.61 ± 0.02^b^	1203	12.11 ± 0.06 ^b^	379	15.96 ± 0.10
Gitilo	1677	2.84 ± 0.01^a^	1238	12.38 ± 0.04 ^a^	411	17.42 ± 0.12
*p-value*		*0.0001*		*0.0003*		*0.0001*
**Season**	**2480**		**2441**		**770**	
Early rain	380	2.72 ± 0.02	377	12.35 ± 0.08 ^a^	119	16.87 ± 0.20
Wet	671	2.73 ± 0.02	659	12.33 ± 0.06 ^a^	241	16.86 ± 0.13
Early dry	987	2.73 ± 0.01	952	12.27 ± 0.05 ^a^	254	16.59 ± 0.12
Dry	442	2.73 ± 0.02	433	12.04 ± 0.07 ^b^	176	16.43 ± 0.15
*p-value*		*0.9382*		*0.0069*		*0.0931*
**Birth Year**	**2480**		**2441**		**770**	
2010	271	2.59 ± 0.03 ^c^	267	12.26 ± 0.10 ^ab^	0	NA
2011	436	2.74 ± 0.03 ^b^	430	12.22 ± 0.07 ^ab^	0	NA
2012	78	2.73 ± 0.0 6 ^abc^	78	12.35 ± 0.17 ^ab^	0	NA
2013	303	2.89 ± 0.02 ^a^	294	11.96 ± 0.09 ^b^	126	17.28 ± 0.19 ^a^
2014	719	2.73 ± 0.01 ^b^	712	12.29 ± 0.07 ^a^	332	16.52 ± 0.11 ^b^
2015	673	2.67 ± 0.02 ^bc^	660	12.39 ± 0.06 ^a^	312	16.270.11 ^b^
2016	7	2.78 ± 0.14 ^ab^	4	13.25 ± 0.63 ^a^	4	15.62 ± 1.00 ^c^
*p-value*		*0.0001*		*0.006*		*0.0001*

Superscripts of different letters in the same column indicate significant differences. NA = not available, LSM = least square mean, SE = standard error, BWt = birth weight, 3MWt = three-month weight, 6MWt = six-month weight.

The mean birth weights of 2.4 kg and 2.43 kg were reported [16] and [17], respectively, for Horro sheep at Debre Berhan Agricultural Research Center (DBARC), which lies outside the ecological niche of the breed. Flocks at Debre Berhan have struggled to adapt to that environment; for instance, the cumulative mortality up to yearling age was more than twice as high for Horro lambs compared to Menz lambs (69.6% vs. 30.2%) [16]. The mean birth weight in the present study was also greater than the 2.15 kg reported for Wollo highland sheep under on-farm conditions [18] and the 2.69 kg for Washera sheep [19] in similar settings. Conversely, the 3.6 kg birth weight of Bonga sheep under on-farm management was higher than the mean weight found in this study [20], likely due to location and breed effects, as [4] noted a high availability of feed in areas where Bonga sheep are raised.

The current study also found a significant (p < 0.001) effect of sex on birth weight. This study was consistent with previous finding of ram lambs averaging 2.76 kg which is heavier than ewe lambs at 2.68 kg [14]. Location significantly influenced lamb birth weight, with higher weights recorded in Gitilo (2.84 kg) compared to Laku Igu (2.61 kg). Given that the breed in both villages is the same, this difference may be attributed to variations in feed availability; Gitilo benefits from extensive communal grazing land, whereas Laku Igu lacks such expansive grazing areas.

Birth year also significantly impacted lamb birth weight (p < 0.001). The likely reason might be due to inconsistencies in the availability of feed, both in quality and quantity. The study indicated that the inconsistency of feed availability results from variations in annual rainfall distribution from 2009 to 2016 [9].

#### Three-month Weight (3MWt).

The overall mean three-month weight of lambs was 12.33 ± 0.03 kg presented in Table 1. All fixed effects had significant influences (at least at p < 0.05) on 3MWt, except the sex of lambs. The absence of a significant effect (p > 0.05) of sex on 3MWt obtained in the current study agrees with other studies [16,17]. Location had a significant influence (p < 0.001) on 3MWt of lambs. The 3Mwt was higher in Gitilo (12.38 ± 0.04 kg) than it was in Laku Igu (12.11 ± 0.06 kg). The likely explanation for the difference in 3MWt between the villages might be related to feed availability. Season of birth had a significant influence on 3MWt. Lambs born in the dry season were significantly (p < 0.05) inferior to those lambs born in other seasons. The dry season is known for a shortage of feed as green feeds are either scarce or not available. This might be because ewes did not get enough amount of feed in their last trimester of pregnancy, where about 65% of the fetal development takes place [21]. Proper feeding during the last phase of pregnancy results in greater development of udder tissues and higher birth weight of lambs. Similarly, other studies also indicate lambs with a heavier birth weight tend to achieve higher weights at weaning, partly because of the positive correlations between traits [9,15,19].

Year of birth significantly (p < 0.01) influenced 3MWt of lambs. The likely reason might be due to the fluctuation of rain across seasons and the year of birth. The result was almost similar with the values 12.2 kg for Horro sheep maintained under on-station management [15] and 12.42 kg for Washera sheep breeds maintained under on-farm management [19]. The 15.5 kg 3MWt reported for Bonga sheep breed managed by members of the Community-based Bonga Sheep breeding program was higher than the current 3MWt reported for Horro sheep flocks [20]. The difference might be related to breed effect and management of the flocks. However, the current value obtained for 3MWt was larger than the 9.48 kg [16] and the 8.2 kg [17] for the same breed evaluated at Debre Berhan Agricultural Research Center (DBARC).

#### Six-month weight (6MWt).

The overall mean of six-month weight obtained in the present study was 16.53 ± 0.07 kg presented in Table 1. This finding was almost similar to the 16.5 kg at on-farm [22], and 16.0 kg at on-station [15] for the same breed under on-farm management systems. However, the 22.2 kg 6MWt reported for Bonga sheep breed [20] and for Horro sheep 18.92 ± 0.50 [10] managed under the CBBP of higher than the value of the current study. Only birth year and location had significant (p < 0.001) influences on 6MWt of lambs. The notable influence of birth year on the 6MWt might be attributed to variations in feed availability, which could result from the distribution and volume of rainfall during that year.

### Genetic parameter estimation for growth traits

Table 2 presents the results of the (co) variance components and genetic parameters for growth traits in Horro sheep.

**Table 2:** Phenotypic correlation (above diagonal), genetic correlation (below diagonal), heritability (on diagonal) for BWt, 3MWt, and 6MWt estimated for Horro sheep under CBBP.

Traits	BWt	3MWt	6MWt
BWt	0.16 ± 0.01	0.01	0.04
3MWt	0.30	0.19 ± 0.01	0.02
6MWt	0.03	0.11	0.28 ± 0.35

BWt = birth weight; 3MWt = three-month weight; 6MWt = six-month weight.

#### Heritability (h^2^) estimates.

The direct additive heritability (h²ₐ) estimate for birth weight (BWt) in this study was 0.16 ± 0.01. This study is greater than the 0.14 for Horro sheep at on-station [14] and 0.14 for Bonga sheep managed under community-based breeding [23]. Moreover, it falls within the range of 0.09 to 0.38 for the Tygerhook Merino sheep breed in South Africa [9]. However, this estimate is lower than the 0.37 reported for the Doyogena sheep managed under community-based breeding [24].

In this study, the estimated h²ₐ value for 3-month weight (3MWt) was 0.19 ± 0.01, which aligns with 0.19 Bonga sheep managed under community-based breeding [23]. However, this estimate is lower than the 0.48 for Menz sheep managed under on-station conditions [25]. The heritability value obtained in this study is also higher than the values ranging from 0.100–0.171 for Barki and Rahmani lambs of Egyptian sheep managed under on-station conditions [26]. These differences may be attributed to variations in breed, location, and seasonal effects.

The h²ₐ estimate for 6-month weight (6MWt) in the current study was 0.28 ± 0.35, which falls within the range of 0.20 ± 0.10–0.49 ± 0.01 of Abera sheep managed under community-based breeding in Ethiopia [27]. It is, however, greater than the 0.16 for Iran-Black sheep [28] and 0.22 ± 0.040 for Bonga sheep managed under community-based breeding in Ethiopia [29]. Moreover, this value is lower than 0.40 reported for Merino sheep [30]. The discrepancies in these estimates may result from differences in sample size, data analysis methods, models used, and breed effects.

#### Genetic and phenotypic correlations.

The genetic (below diagonal) and phenotypic correlation (above diagonal) presented in Table 2. In this study, low to medium genetic correlations among growth traits were observed. The genetic correlations between BWt and 3MWt, BWt and 6MWt, and 3MWt and 6MWt were 0.30, 0.03, and 0.11, respectively. The correlation between BWt and 3MWt was moderate. These genetic correlation values are lower than the 0.45, 0.33, and 0.93 reported for the same traits of the same sheep breed maintained at the Bako Agricultural Research Center [14].

The estimated phenotypic correlations between BWt and 3MWt, BWt and 6MWt, and 3MWt and 6MWt were 0.01, 0.04, and 0.02, respectively. These estimates differ from the 0.25, 0.20, and 0.73 reported for the same traits [14]. The authors utilized on-station data collected over two decades and employed a variety of models, while this study used only a simple animal model.

### Genetic trends

**Fig 1:**
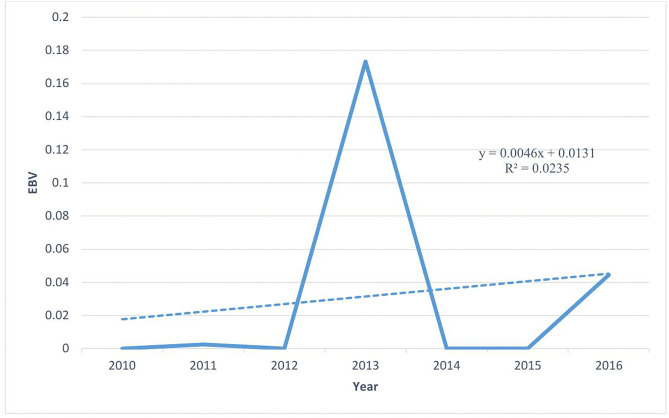
Genetic trend for birth weight (BWt) based on annual mean estimated breeding values (EBVs) from 2010 to 2016.

The genetic trends for weight at birth (BWt), three-month weight (3MWt), and six-month weight (6MWt) over the years showed an upward trajectory, indicating positive genetic trends. The reason for an upward trajectory might be due to the selection of breeding rams. Consequently, the success of a breeding program can be evaluated by the actual change in breeding value expressed as a proportion of the expected theoretical change in breeding value for the trait under selection. Genetic trends were estimated by regressing annual mean EBVs on year. The estimated genetic gain for BWt was + 0.0046 kg per year during 2010–2016, with the trend in mean EBVs illustrated in Fig 1.

**Fig 2:**
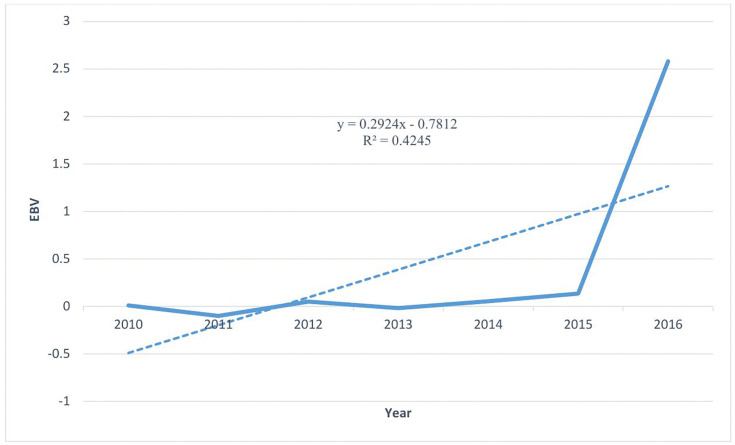
Genetic trend for three-month weight (3MWt) based on annual mean estimated breeding values (EBVs) from 2010 to 2016.

Similarly, positive genetic trends were observed for 3MWt and 6MWt, further confirming improvement in growth performance. The estimated genetic gain for 3MWt was + 0.2924 kg per year over the same period, and the corresponding EBV trend is presented in Fig 2. This study is in alignment with research conducted on the Menz sheep breed managed under a Community-Based Breeding Program. The findings indicated a positive phenotypic improvement trend of 0.417, 0.408, 0.427, and 0.160 kg over the years in the Dargegn, Molale, Sinamba, and Zeram villages, respectively [31]. Although those Menz’s sheep breed estimates represent phenotypic improvement, the consistent upward trend supports overall progress in growth performance under community-based breeding schemes.

**Fig 3:**
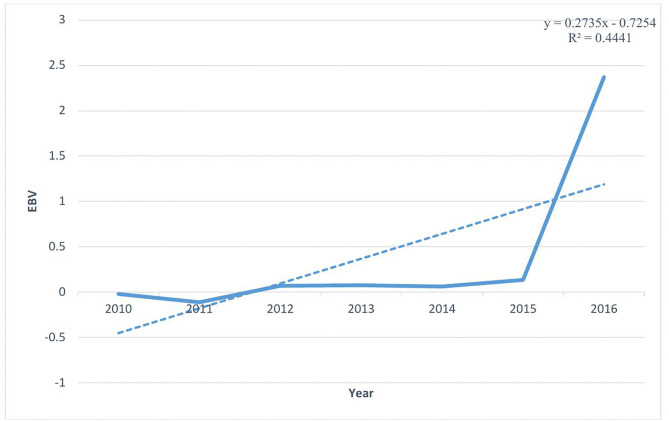
Genetic trend for six-month weight (6MWt) based on annual mean estimated breeding values (EBVs) from 2010 to 2016.

Weight at six months is a targeted trait for improvement in the CBBP [3,5,7], also exhibited a favorable genetic trend. The estimated genetic gain for 6MWt was + 0.2735 kg per year during 2010–2016, with the mean EBVs illustrated in Fig 3. These results suggest that continued and more structured selection could further enhance genetic progress in growth traits.

However, the CBBP faces challenges in managing breeding rams, particularly in their rotation [32]. The genetic improvement program stipulates that a breeding ram should be used for only one year in a specific area before being transferred to other community members who share grazing land and watering points [3,5,7]. Nevertheless, researchers have observed that most breeding rams remain with the flocks in which they were raised, lacking structured management and usage [10].

## Conclusion

The significant effects of fixed factors underscore the necessity of incorporating them into the design of genetic improvement strategies for the sheep breed. The moderate heritability indicates that genetic enhancement can be realized through the selection of sheep beginning in early growth stages. The trends in genetic improvement over the years demonstrate the success of the established breeding program. Consequently, to enhance the effectiveness of community-based breeding programs beyond the observed changes, it is recommended that efforts be made to include proper record-keeping and the selection of rams based on birth weight, 3-month weight, and 6-month weight.
